# Dynamics of different-sized solid-state nanocrystals as tracers for a drug-delivery system in the interstitium of a human tumor xenograft

**DOI:** 10.1186/bcr2330

**Published:** 2009-07-03

**Authors:** Masaaki Kawai, Hideo Higuchi, Motohiro Takeda, Yoshio Kobayashi, Noriaki Ohuchi

**Affiliations:** 1 Division of Surgical Oncology, Tohoku University Graduate School of Medicine, Tohoku University, 1-1 Seiryo-machi, Aoba-ku, Sendai, 980-8574, Japan; 2 Department of Physics, Graduate School of Science, The University of Tokyo, 7-3-1 Hongo, Bunkyo-ku, 113-0033, Japan; 3 Department of Nano-Medical Science, Graduate School of Medicine, Tohoku University, 1-1 Seiryo-machi, Aoba-ku, Sendai, 980-8574, Japan; 4 Department of Biomolecular Functional Engineering, College of Engineering, Ibaraki University, 4-12-1 Naka-narusawa-cho, Hitachi, 316-8511, Japan

## Abstract

**Introduction:**

Recent anticancer drugs have been made larger to pass selectively through tumor vessels and stay in the interstitium. Understanding drug movement in association with its size at the single-molecule level and estimating the time needed to reach the targeted organ is indispensable for optimizing drug delivery because single cell-targeted therapy is the ongoing paradigm. This report describes the tracking of single solid nanoparticles in tumor xenografts and the estimation of arrival time.

**Methods:**

Different-sized nanoparticles measuring 20, 40, and 100 nm were injected into the tail vein of the female Balb/c nu/nu mice bearing human breast cancer on their backs. The movements of the nanoparticles were visualized through the dorsal skin-fold chamber with the high-speed confocal microscopy that we manufactured.

**Results:**

An analysis of the particle trajectories revealed diffusion to be inversely related to the particle size and position in the tumor, whereas the velocity of the directed movement was related to the position. The difference in the velocity was the greatest for 40-nm particles in the perivascular to the intercellular region: difference = 5.8 nm/s. The arrival time of individual nanoparticles at tumor cells was simulated. The estimated times for the 20-, 40-, and 100-nm particles to reach the tumor cells were 158.0, 218.5, and 389.4 minutes, respectively, after extravasation.

**Conclusions:**

This result suggests that the particle size can be individually designed for each goal. These data and methods are also important for understanding drug pharmacokinetics. Although this method may be subject to interference by surface molecules attached on the particles, it has the potential to elucidate the pharmacokinetics involved in constructing novel drug-delivery systems involving cell-targeted therapy.

## Introduction

The strategies of recently developed cancer therapy are designed to deliver sufficient doses of drugs to the target tumor and to reduce any unnecessary damage to the normal organs. The effects of numerous drugs have been maximized by controlling their diameter to appropriate sizes to pass selectively through the tumor vasculature. Tumors contain a high density of dilated vessels with poor architecture [1], which have larger pores than normal vessels and exhibit higher permeability [2]. This characteristic contributes to higher concentrations of plasma macromolecular proteins in the tumor interstitial space [3-6]. Once a drug is administered, it passes through the vascular pores and thereafter must overcome interstitial and intercellular barriers to approach malignant cells. Interstitial drug transport is determined by both diffusion and the directional flow, with a net flow of fluid from blood vessels to the lymphatic system [7] influenced by components of the tumor-specific extracellular matrix, notably collagens and proteoglycans [8]. Large drugs in the interstitium are transported more slowly [9] and thus are retained in the interstitial space for longer periods [10]*in vivo *because the interstitial matrix blocks drug movement [11,12]. The size dependency of *in vivo *drug delivery in the tumor interstitium has been quantitatively estimated by observing the average dispersion of drugs [13]. However, these general observations cannot fully explain the behavior of individual drugs, such as their binding to the interstitial matrix and nonuniform movements. Conversely, single-particle tracking is a powerful method for gaining unprecedented reductive information regarding individual drug molecules, such as molecular motion in biophysics [14,15] and the delivery processes of antibodies [16].

The present study focused on single-particle tracking to elucidate the influence of homogeneous size on the movement of the nanoparticles in tumor xenograft. The sizes of the nanoparticles for drug-delivery systems were optimized in relation to the molecular size of the agent. Hence, the delivery processes were clarified, and then these processes were quantitatively analyzed to clarify the rate-limiting constraints for single-nanoparticle delivery in the interstitial space *in vivo*.

## Materials and methods

### Fluorescent beads and Quantum dot 705

"Fluospheres" were manufactured from high-quality ultraclean polystyrene microspheres by Invitrogen Molecular Probes (Eugene, OR, USA). Fluospheres [17] and Quantum dot 705 ITK kit (Quantum Dot Corp., Hayward, CA, USA) were useful as markers during observations because of their intense brightness. Quantum dot 705 was used for 20-nm nanoparticles. Fluospheres of 40, 100, and 200 nm were selected, emitting orange fluorescence (excitation/emission maxima at 540/560 nm). The diameter distributions of the beads were very small at 100 ± 5 nm (<5% error) and 200 ± 10 nm (<5% error). The zeta potentials, electricity of the inner area, and the conceptual surface of Quantum dot, Fluospheres (40 nm and 100 nm) are -4.97 mV, -18.60 mV, and -33.73 mV, respectively.

### Cell line

The human breast cancer cell line KPL-4 [18] was kindly provided by Dr. J. Kurebayashi (Kawasaki Medical School, Kurashiki, Japan). KPL-4 cells were cultured in Dulbecco's Modified Eagles Medium (DMEM) supplemented with 5% Fetal Bovine Serum (FBS).

### Mouse model

A suspension of KPL-4 cells (1.0 × 10^7 ^cells/100 μl DMEM) was subcutaneously transplanted into the dorsal skin of female Balb/c nu/nu mice at 5 to 7 weeks of age (Charles River Japan, Yokohama, Japan). Several weeks after the inoculation, mice with tumor volumes of 100 to 200 mm^3 ^were selected. All mice were maintained in pathogen-free facilities. All operations were carried out in accordance with the Institutional Animal Use and Care Regulations of Tohoku University, after receiving approval from the Committee on Animal Experiments.

The mice were anesthetized with an intraperitoneal injection of a mixture of ketamine and xylazine at doses of 95 and 5 mg/kg, respectively. The temperature of the mice was maintained at 37°C by using an objective lens heater.

After imaging, the mice were killed with a CO_2 _overdose. The tumors were removed and divided for histologic nanoparticle-uptake studies 24 hours after the administration of the particles. The tumors were fixed in optimal cutting temperature (OCT) compound (Sakura Finetek, Torrance, CA, USA), frozen, cut into 6-μm sections, and either examined with confocal microscopy or stained with hematoxylin and eosin and examined with bright-field microscopy.

### Dorsal skin-fold chambers

Dorsal skin-fold chambers, which were described in a previous study [19,20] and modified for the present study, were used to fix the mouse tumors over the objective lens of an inverted microscope [see Additional data file 1]. A sterilized polyvinyl chloride plate with a small window was mounted on the stage to fix the extended double layer of dorsal skin, including the tumor. The skin was sutured with 5-0 nylon around the window and fixed. A spatial accuracy of 30 nm was achieved. The beating of the heart and breathing have a serious influence on this measurement. It was, therefore, necessary to cancel the heartbeat and breathing by fixing. The tumor was exposed with oval skin and subcutaneous incisions of ~10 mm in diameter and then placed on a neutral saline-mounted coverslip (0.12 to 0.17 mm in thickness) on the viewing platform of the microscope. The mouse was fixed on the stage to stabilize the chamber [see Additional data file 2]. The tumors were thereafter directly visualized by using this set-up.

### In vivo imaging and tracking

The optics system for the observations consisted primarily of an epifluorescence microscope (IX71; Olympus, Tokyo, Japan) with modifications [21,22], a Nipkow lens confocal unit (CSU10; Yokokawa, Tokyo, Japan), and an electron multiplier charge-coupled device (EMCCD) camera (iXon 887; Andor, Tokyo, Japan) [see Additional data file 2]. The confocal unit adopts multibeam scanning by using about 1,000 beams that are simultaneously emitted through a pinhole disk to facilitate high-speed scanning. The EMCCD has the advantage of offering unsurpassed sensitivity and has been shown to yield markedly improved signal-to-noise ratios [23]. The objective lens (×60; NA, 1.45) was moved by a piezo actuator with a feedback loop (Nano Control, Tokyo, Japan) to stabilize the position of the focus. A computer controlled the piezo actuator in synchronization with the image acquisitions, such that the objective lens remained within the exposure time of the EMCCD camera. An area of ~30 × 30 μm^2 ^was illuminated by a green laser (532 nm; Crystalaser, Reno, NV, USA). This system can capture images of a single nanoparticle at a video rate of 33 ms/frame.

The xy position of each fluorescent spot was calculated by fitting to a two-dimensional gaussian curve. Single molecules could be identified by their fluorescence intensities. The accuracy of the x and y directions of images taken at an exposure time of 33 ms was 30 nm, while also taking the standard deviation into consideration.

### Calculations of the mean square displacements, diffusion coefficients

The trajectories were calculated by using MATLAB 6.0 Release 12 (The MathWorks Inc., Natick, MA, USA). The mean square displacements (MSDs) of individual nanoparticles were defined by the following equation:



where *x*_*i *_and *y*_*i *_are the positions on frame *i*, *N *is the total number of frames, Δ*t *is the time between frames and distance between steps in time *t *[24], and *n*Δ*t *is the time interval over which the MSD is calculated. When the change in the MSD was nonlinear over time, the curve could be fitted by the equation for confined diffusion [24,25]. For direct diffusion, in which a nanoparticle moves in a direction at a constant drift velocity with diffusion, the MSD plot is convex and closer to time 0, where the change in the MSD was linear. Single-molecule diffusion was considered to be brownian motion, and the diffusion coefficient (D), representing the amount of substance diffusing across an area through a unit concentration gradient in unit time, was calculated according to the following equations:





### Statistical analysis

All statistical analyses were carried out by using the StatMate III software program (ATMS Co. Ltd., Tokyo, Japan). The MSDs, velocities, and diffusion coefficients for the three different-sized particles were compared with a two-way analysis of variance (ANOVA). The correlations between the velocity, diffusion coefficient, and position for the same particle were determined by using the Spearman rank test. All probabilities (*P *values) of the statistical tests were based on two-tailed tests, and values of *P *< 0.05 were considered to indicate statistical significance.

## Results

### Imaging of normal and tumor vasculatures

**Figure 1:**
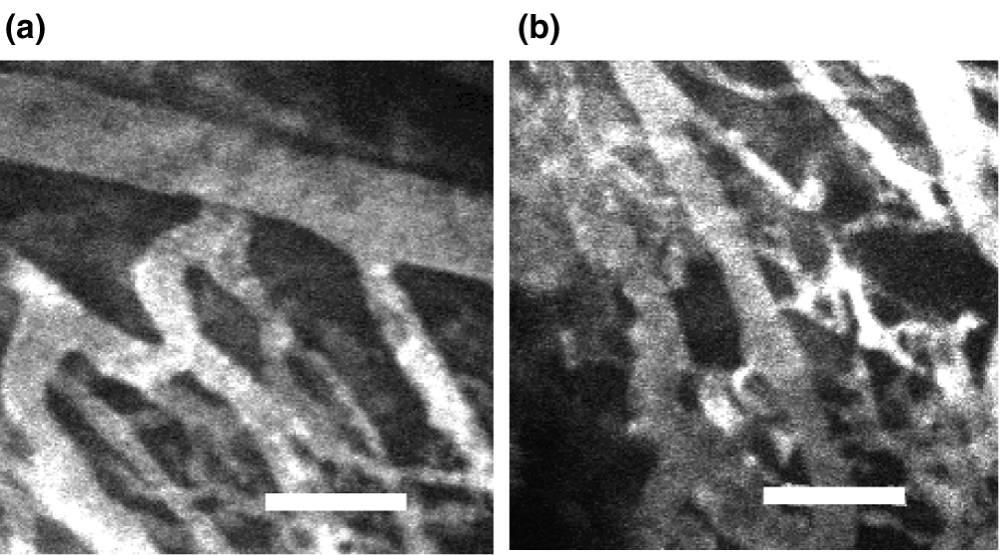
Normal and tumor vasculatures visualized by intravital imaging. An objective lens of 20× was used in this experiment. **(a) **Normal vessels are aligned parallel to each other. **(b) **Tumor vessels have chaotic characteristics, such as uneven diameters and unparallel distribution. Scale bar, 100 μm.

To visualize and confirm the macroscopic characteristics of normal and tumor vasculatures, Quantum dot 705 (40 nmol/l in 200-μl phosphate-buffered saline; PBS) was administered into the tail vein of mice with human breast cancer xenografts and observed with intravital confocal microscopy. In the normal vasculature, the blood vessels were parallel and straight relative to one another (Figure 1a). In the tumor vasculature, however, the blood vessels were chaotically disorientated with uneven sizes (Figure 1b). A typical example of the pathologic nature of the tumor vasculature is illustrated by the looped chaotic structure in the center of Figure 1b[26].

### Histopathologic analysis of extravasation of single nanoparticle in tumor xenograft in mice

**Figure 2:**
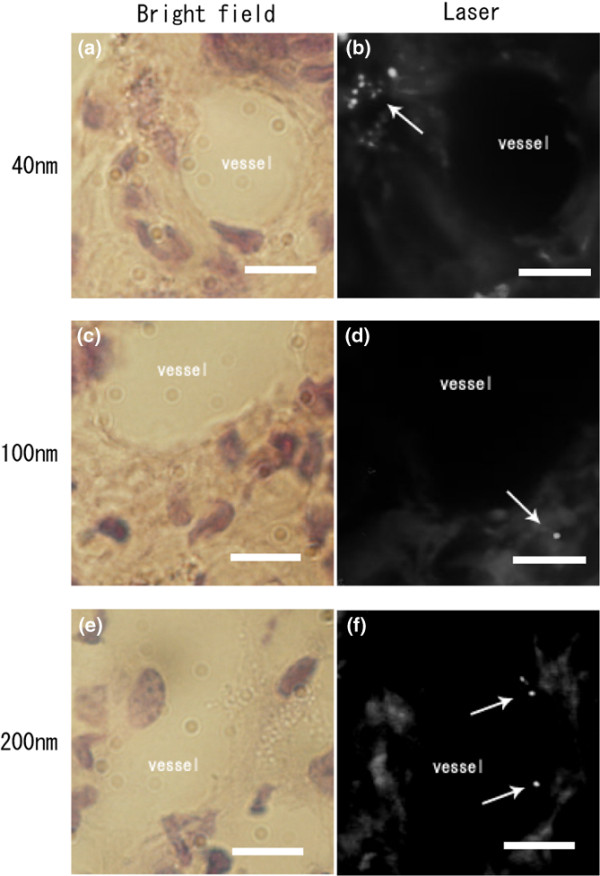
Histologic evaluation of extravasation of each nanoparticle. Extravasation of single nanoparticles in mice with human tumor xenografts. **(a, c, e) **Hematoxylin and eosin-stained images observed by bright-field microscopy after injection of 40-, 100-, and 200-nm particles, respectively. **(b, d, f) **Laser confocal microscopy images after injection of 40-, 100-, and 200-nm particles, respectively. Particles of 40 nm (b) and 100 nm (d) extravasated (arrows), whereas 200-nm nanoparticles did not extravasate (arrow) (f). Scale bar, 10 μm.

After the injection of nanoparticles, the tumors were fixed, cut into 6-μm sections, and either examined with confocal microscopy or stained with hematoxylin and eosin and examined with bright-field microscopy. Images of the tumors were taken to allow observation of the nanoparticles in the tumor vessels and interstitium (Figure 2a–f). Particles of 40 and 100 nm were present in the interstitial region (arrows in Figure 2b and 2d, respectively) in 20 visual fields. In contrast, 200-nm particles were found only in the vessels (arrows), but not in the interstitial region (Figure 2f).

### Trajectories of single nanoparticles in perivascular, interstitial and intercellular areas

The injected nanoparticles circulated within the tumor vessels. Some of the nanoparticles became extravasated and moved into the perivascular area close to the tumor vessels. The vessel boundaries were traced by accumulating all the acquired images and were superimposed onto the actual trajectories of the nanoparticles.

**Figure 3:**
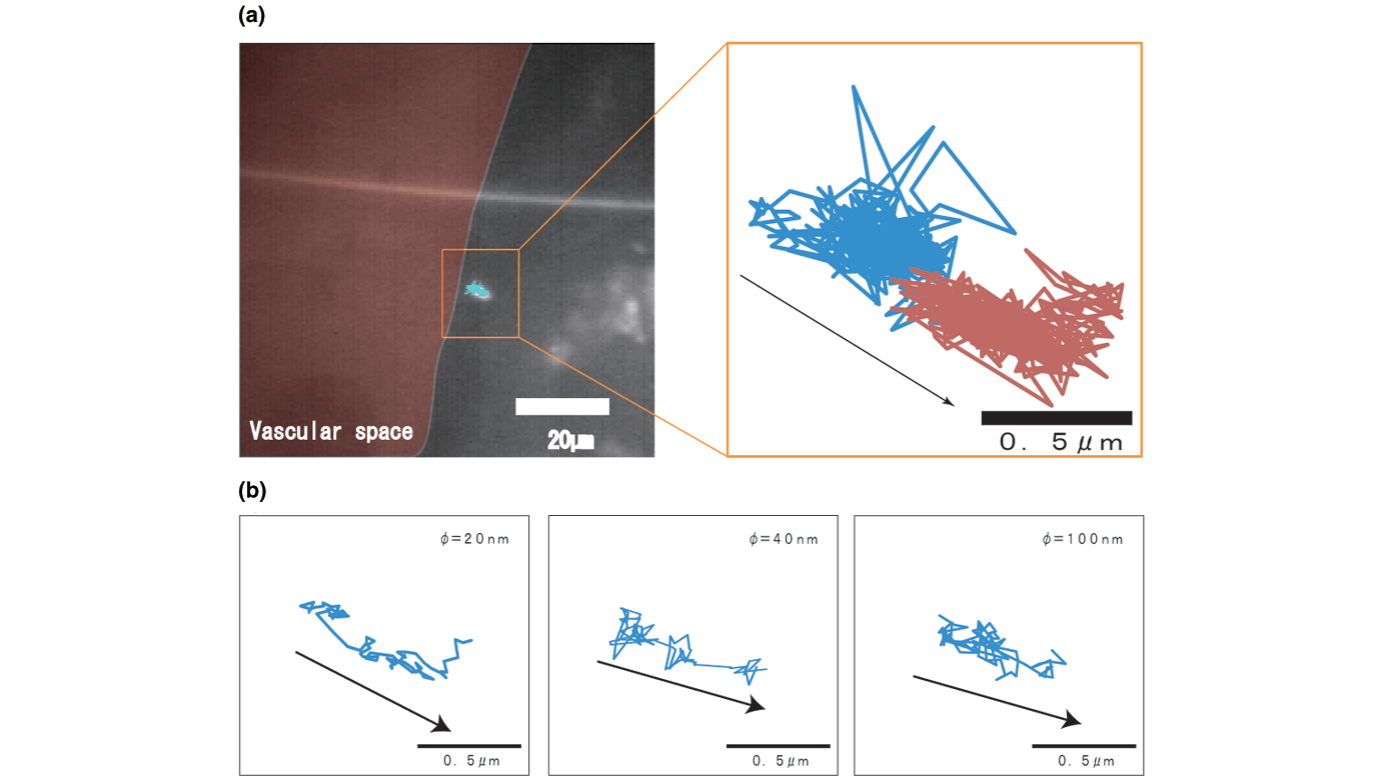
Trajectories of nanoparticles in the perivascular region. The arrow indicates the direction of the movements of the nanoparticles. **(a) **Trajectories of the Quantum dot in the perivascular region. The trajectories are encoded on the time axis from blue to red. **(b) **Trajectories of three different-sized particles in the perivascular area.

Suddenly after the injection, the nanoparticles appeared in the tumor vasculature. As shown in Figure 3a, a 20-nm particle was found to diffuse within a restricted area (~800 nm in diameter) and then suddenly move to the next point. The acquired trajectories of single particles of different sizes in the perivascular area are shown in Figure 3b. For 20-nm particles, the area of random diffusion composed only a small part of the overall trajectory, whereas directed movement occupied the main part. However, larger molecules showed increased random diffusion.

**Figure 4:**
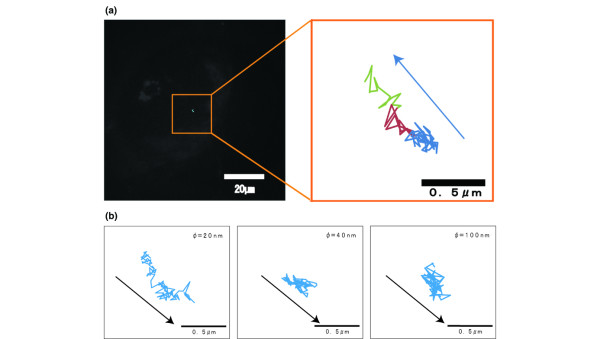
Trajectories of nanoparticles in the interstitial region. **(a) **Trajectories of the Quantum dot in the interstitial region. The trajectories are encoded on the time axis from blue to red to yellow. **(b) **Trajectories of three different-sized particles in the interstitial region.

At 2 hours after the injection, the migrated nanoparticles were tracked in the tumor interstitial region. As shown in Figure 4a, a 20-nm particle diffused within a highly restricted area, although it appeared to be smaller than in the perivascular region. In Figure 4b, diffusion contributed a much larger part of the movement for 20-nm particles. For 40- and 100-nm particles, the movement appeared to be diffusional.

**Figure 5:**
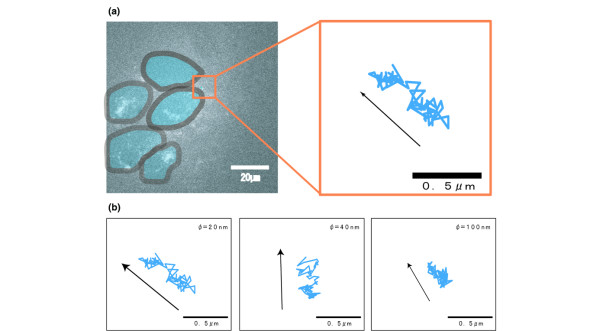
Trajectories of nanoparticles in the intercellular region. **(a) **Trajectories of the Quantum dot in the intercellular region. **(b) **Trajectories of three different-sized particles in the intercellular region.

About 3 hours after the injection, the nanoparticles were seen in the tumor intercellular region. The cell boundaries were traced by accumulating all the acquired images and were superimposed onto the actual trajectories. As shown in Figure 5a, a 20-nm particle movement appeared to be much smaller. In Figure 5b, the proportion of diffusion of particles was increased. The movements of 40- and 100-nm particles appeared to represent only diffusion, indicating that these particles are under brownian motion.

### Mean square displacements, velocities, and diffusion coefficients in different regions of tumors

**Figure 6:**
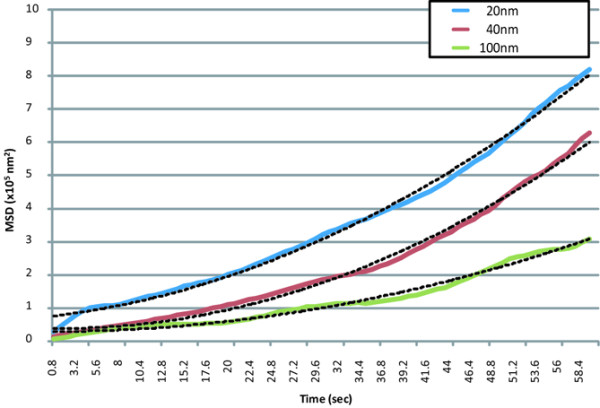
MSD plots of nanoparticles in the perivascular regions. Data represent the means (solid lines) and two-dimensional fitted curves (dotted lines) of particles.

The MSD was calculated by the positional data for each particle. The MSD plots of the particles in the perivascular, interstitial, and intercellular regions are shown in Figures 6 through 8, respectively.

**Figure 7:**
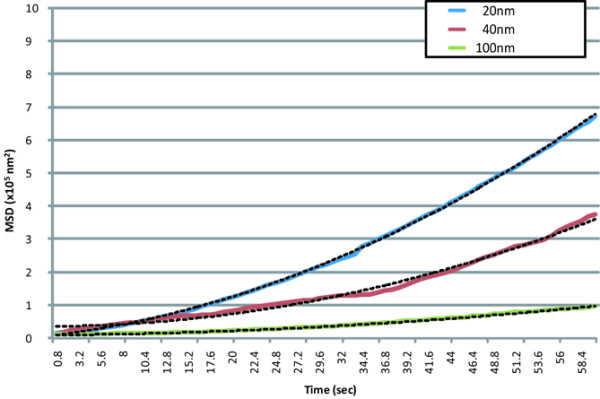
MSD plots of nanoparticles in the interstitial regions. Data represent the means (solid lines) and two-dimensional fitted curves (dotted lines) of particles.

**Figure 8:**
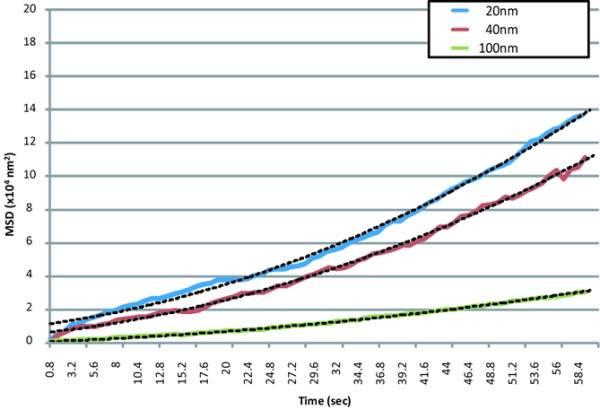
MSD plots of nanoparticles in the intercellular regions. Data represent the means (solid lines) and two-dimensional fitted curves (dotted lines) of particles.

The movement plots of the different-sized molecules in the perivascular region were convex. The movements of each molecule were well fitted to curves, assuming directed movement made by lymphatic flow from vasculature to lymphatic system (Figure 6) with superimposed random diffusion [24] but not fitted to either simple directed movement or random diffusion. The *R*^2 ^values of the fitted equations (dotted lines) were 0.98, 0.99, and 0.97 for 20-, 40-, and 100-nm particles, respectively. In the interstitial region, the plots for the different-sized molecules were also convex, which was similar to those in the perivascular region, but the steepness of each of the three curves was lower than the corresponding curve in the perivascular region. The *R*^2 ^values of the fitted equations were 1.00, 0.98, and 0.98 for 20-, 40-, and 100-nm particles, respectively. The apparent values of the MSD plots in the intercellular region were approximately half or one order of magnitude lower than those in the other two regions. The *R*^2 ^values of the fitted equations (two-dimensional) were 0.97, 0.97, and 0.94 for 20-, 40-, and 100-nm particles, respectively.

**Figure 9:**
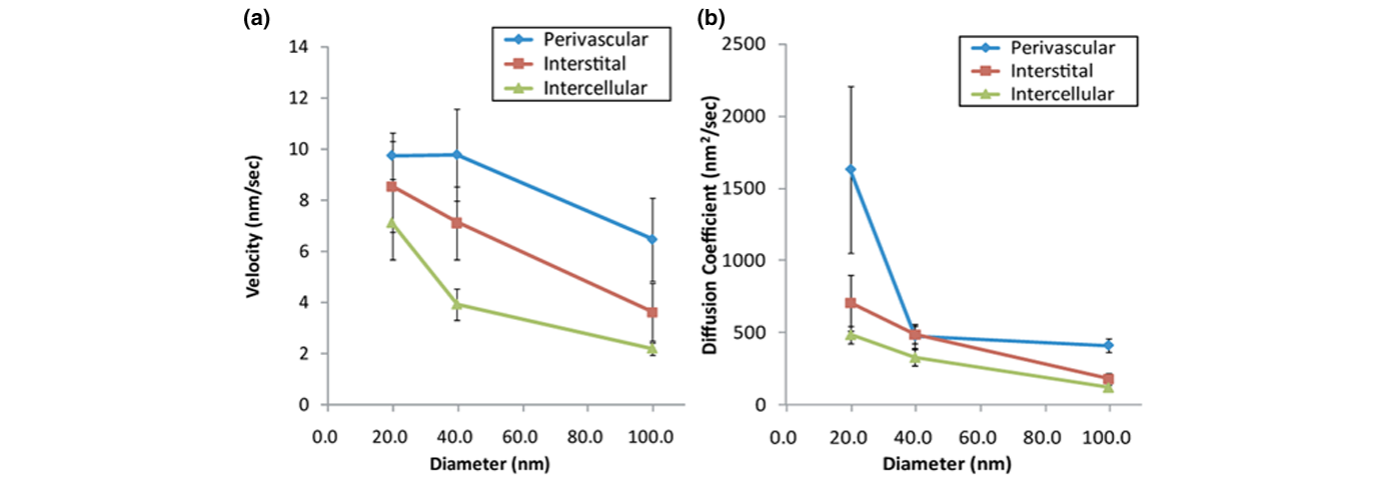
Velocities and diffusion coefficients in different regions of tumors. **(a) **Velocities relative to the particle diameters in different regions of tumors. **(b) **Diffusion coefficients relative to the particle diameters in different regions of tumors. Blue line: 20-nm; red line: 40-nm; green line: 100-nm, respectively.

The relations were analyzed between the velocities and diffusion coefficients and the particle diameters and positions in the tumor (perivascular, interstitial, and intercellular regions). As shown in Figure 9a, the velocity changed in relation to the particle position. Notably, the difference in velocity was greater for 40-nm particles (from 9.7 nm/s in the perivascular region to 7.1 nm/s in the interstitial region and 3.9 nm/s in the intercellular region) than for 20- and 100-nm particles and negatively correlated with the position (correlation coefficient = -5.5; *P *< 0.001, Spearman rank test). The diameters of the particles were not related to the velocities in the perivascular and interstitial regions (n = 6, *P *= 0.39 for the perivascular region and *P *= 0.14 for the interstitial region (two-way ANOVA test) but were related to the velocities in the intercellular region (n = 6; *P *= 0.016, two-way ANOVA test). All three curves were parallel, indicating an inverse relation with the diameter at intercellular region. As shown in Figure 9b, the diffusion coefficients of the three curves were convex, inversely related to the diameter. Notably, a steep difference was found between the 20- and 40-nm particles in the perivascular region (correlation coefficient = -0.4; *P *< 0.001, Spearman rank test). For 20-nm particles, a remarkable disparity was seen between the particle positions, especially in the perivascular and interstitial regions. The diameters of the molecules in the perivascular and interstitial regions were related to the diffusion coefficients (n = 6; *P *< 0.05 for two regions; two-way ANOVA test).

## Discussion

The present study succeeded in clarifying the specific delivery processes of single nanoparticles after injection into the mice [16], as well as tracking and quantifying the detailed movements of different-sized nanoparticles in the interstitium of tumor xenograft. We clarified the specific behavior of the solid-sized nanoparticle and the relations among the size, place, velocities, and diffusion for the individual mechanisms in the interstitial space for the first time at the single-particle level.

The structural characteristics of the tumor vasculature are strongly related to the function of neovascularized tumor vessels, because antiangiogenic agents have the potential to normalize the tumor vasculature and to change the efficiency of drug delivery [27]. The tumor vasculature of KPL-4 cancer cells was visualized, because the characteristics of their neovascularized vessels have not yet been clarified. Quantum dot was used as the contrast agent because it has a stronger size effect, thus enabling it to stay in the vessels for longer periods than low-molecular-weight agents [28].

In previous studies, the movements of macromolecules have been analyzed *ex vivo *[29] and *in vivo *[9]. These studies made it possible to visualize several aggregated particles, but not single particles. Conversely, single-particle tracking has recently been applied to mice [16]. Our results regarding the relation between the drug size and movements with single-particle tracking are important to design recently manufactured nanocapsules such as polymeric micelles [30], liposomes [31], and dendrimers [32]. These nanocapsules stay and show their effect on the targeted tumor in a single particle, and thus we can measure the precise point where these particles show effects only with our single-particle tracking, not by the previously mentioned method. Furthermore, the sizes of these drugs are changeable by manufacturing procedures; we can design their diameter to control the time that the drug shows its effect on the tumor (we mention the calculated time in the last paragraph in Discussion). Single-particle tracking is therefore considered to be important for the design of these drugs.

The movement of particles in each process was the sum of random diffusions within a highly restricted area and directed movements toward another area. These data show that the velocities of the three different-sized particles decreased while moving in the three regions in a parallel manner. The diffusion coefficients were more significantly decreased according to size in the perivascular region than in the other two regions. It is generally thought that the tumor interstitium consists of a collagenous cage structure containing viscous proteoglycans [8] and that these matrices thus block drug movement [11,12]. The data for the particle velocities are consistent with previous data showing the velocity to be weakly dependent on the particle size [8] and dependent on the decreasing interstitial flow from the perivascular region [33,34] and that the diffusion coefficients of particles are limited in proportion to the particle sizes [35]. Others studies have shown that the extracellular matrix collagen content is not related to particle speed [35] but does restrict particle diffusion. The current data appear to indicate that directional movement is disrupted by the particle position, rather than the particle size, because the three-dimensional structure of the collagenous matrix was sufficiently loose to allow movements of the different-sized particles and free diffusion of the 20-nm particles in the perivascular region, but limited the diffusion of particles larger than 20 nm because of the presence of viscous hyaluronan.

Other vesicles, such as polyethylene glycolated (PEGylated) materials, are used as drug carriers, but they are associated with problems in drug release because of their stable structure; they also contain releasing drugs in themselves. The solid-state carriers used in the current study can be made functional by attaching molecules. Furthermore, the accumulation of the conjugation and uptake of the Quantum dot-antibody complex in the tumor [16] could be detected. The particle used in the present study can therefore be individually detected, and the pore size can be estimated because of its solidity. The time required for a drug to reach the tumor is an important issue for measuring the time to show its effect. We used these data to simulate the time required for a single agent to reach a tumor cell after extravasation. Because many anticancer drugs affect cancer cells at mitosis, 78.9 μm was selected as the average distance between mitotic cells and their adjacent microvessels in the tumor [36]. The perivascular, interstitial, and intercellular regions were assumed to be equally distributed. After extravasation, the estimated times for different particles of the 20-, 40-, and 100-nm particles to reach the tumor were 140.0, 196.9, and 358.4 minutes for 2.25% of the particles; 158.0, 218.5, and 389.4 minutes for 50% of the particles; and 178.5, 242.6, and 423.1 minutes for 97.5% of the particles, respectively.

## Conclusions

Elucidating the precise mechanism for interstitial movement of nanoparticles in animal models of human breast cancer will clarify the fundamental aspects of the drug-delivery process. The method used in the present study could therefore be potentially useful for drug design to increase the effectiveness of tumor-targeting nanoparticles.

## Abbreviations

ANOVA: analysis of variance; DMEM: Dulbecco's Modified Eagles Medium; EMCCD: electron multiplier charge-coupled device; FBS: fetal bovine serum; MSD: mean square displacement; OCT: optimal cutting temperature; PBS: phosphate-buffered saline; PEG: polyethylene glycol.

## Competing interests

Noriaki Ohuchi has received research grants from Takeda Pharmaceutical Company Limited, and Konica Minolta Medical & Graphic, Inc. Motohiro Takeda has received a research grant from Konica Minolta Medical & Graphic, Inc. Masaaki Kawai, Hideo Higuchi, and Yoshio Kobayashi have no competing interests.

## Authors' contributions

MK, HH, and MT conceived of and designed the research. MK, HH, MT, YK, and NO drafted the manuscript. MK, HH, and YK performed the research. MK and HH contributed new reagents or analytic tools. MK, HH, and YK analyzed the data. All authors read and approved the final manuscript.

## Supplementary Material

Additional file 1 Picture of a dorsal skin-fold chamber. The skin between the chambers is sutured with 5-0 nylon around the window to locate the tumor in the center of the window. The tumor is exposed by incisions and then placed on a coverslip on the microscope. Click here for file

Additional file 2 Scheme of the optic system. It consists of an epifluorescence microscope, a Nipkow lens confocal unit, and an EMCCD camera. Click here for file
