# Supplementary figures and images for: Dynamics of different-sized solid-state nanocrystals as tracers for a drug-delivery system in the interstitium of a human tumor xenograft
Source: Breast Cancer Res. 2009 Jul 3;11(4):R43. doi: 10.1186/bcr2330 (PMC2750102; doi:10.1186/bcr2330)

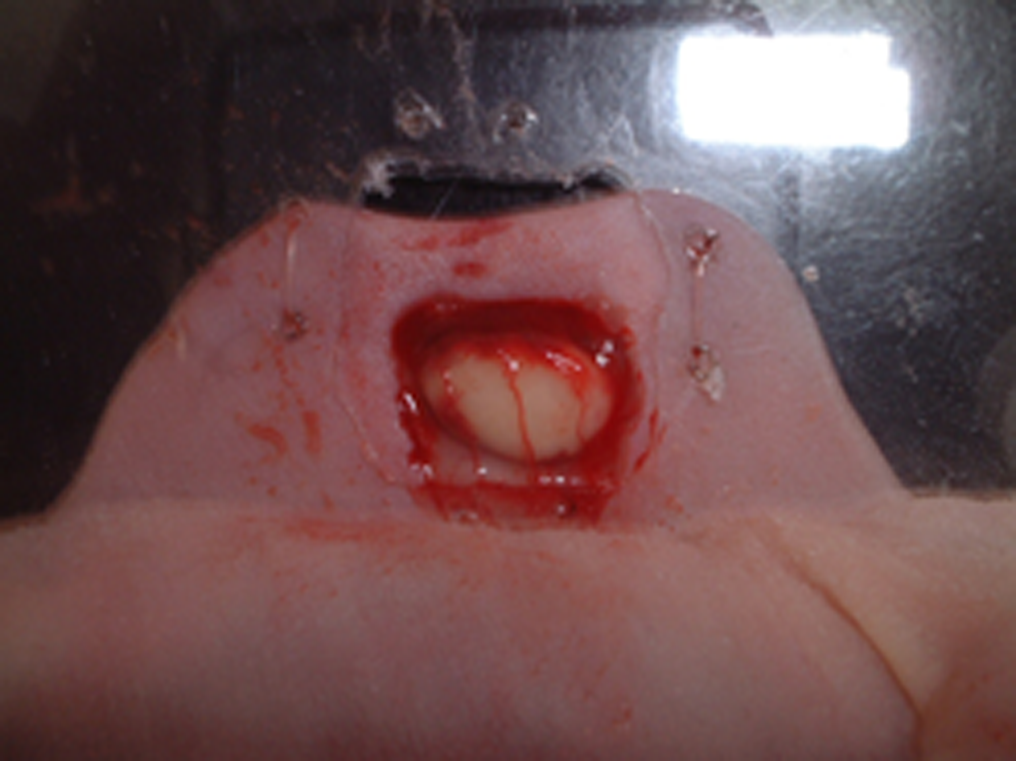

Supplement: Additional file 1 — Picture of a dorsal skin-fold chamber. The skin between the chambers is sutured with 5-0 nylon around the window to locate the tumor in the center of the window. The tumor is exposed by incisions and then placed on a coverslip on the microscope. [file bcr2330-S1.tiff]

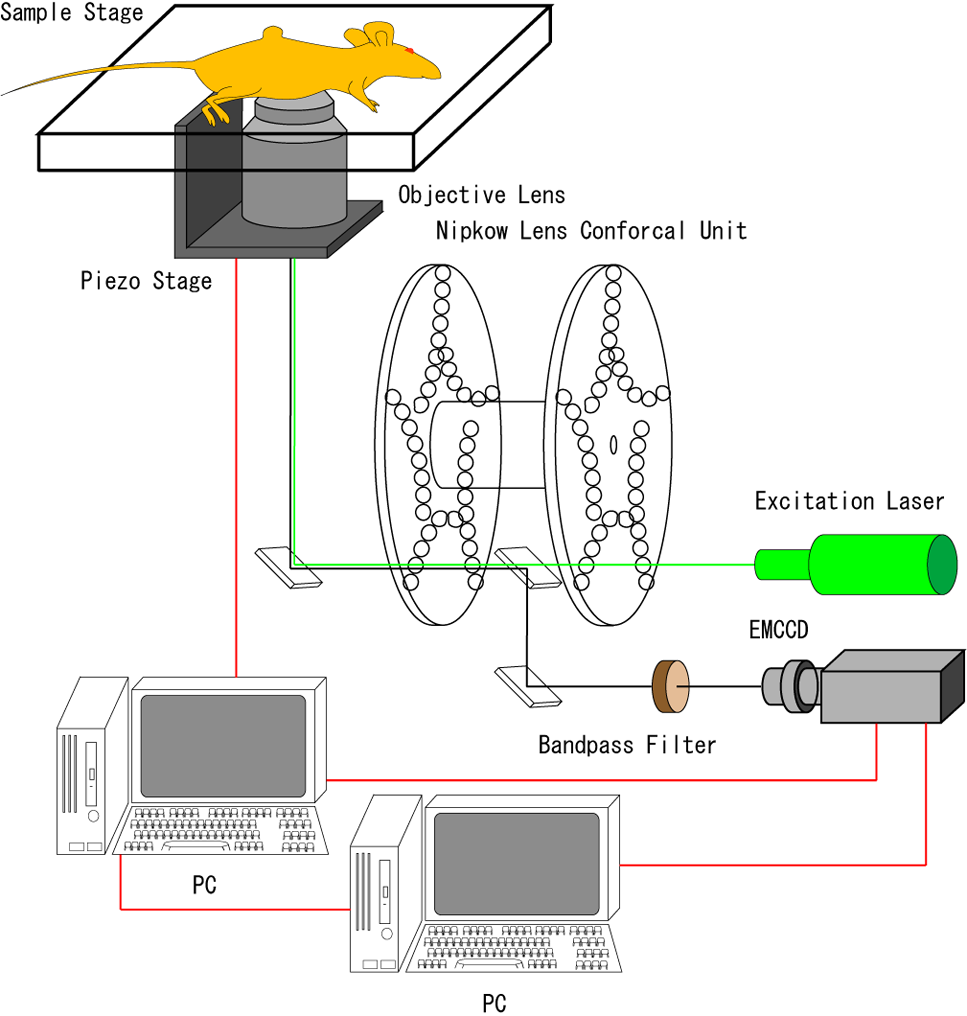

Supplement: Additional file 2 — Scheme of the optic system. It consists of an epifluorescence microscope, a Nipkow lens confocal unit, and an EMCCD camera. [file bcr2330-S2.tiff]
